# Early childhood blood pressure trajectories in very low birth weight offspring: is there a legacy of maternal hypertension?

**DOI:** 10.1016/j.jped.2025.101482

**Published:** 2025-12-18

**Authors:** Daiane de Oliveira Pereira Vergani, José Mauro Madi, Lucas Girotto de Aguiar, Vitória Rovatti Canello, Thiago Crocoli Balbinot, Letícia Lorenzet, Luciano da Silva Selistre, Vandréa Carla de Souza

**Affiliations:** a Universidade de Caxias do Sul, Programa de Pós-Graduação em Ciências da Saúde, Caxias do Sul, RS, Brazil; b Universidade de Caxias do Sul, Área do Conhecimento de Ciências da Vida, Caxias do Sul, RS, Brazil; c Hospital Geral de Caxias do Sul, Caxias do Sul, RS, Brazil

**Keywords:** Hypertensive disorders of pregnancy, Blood pressure, Preeclampsia, Preterm birth, Very low birth weight, Offspring

## Abstract

**Objective:**

To investigate the association between maternal hypertensive disorders of pregnancy (HDP) and blood pressure (BP) in preterm children born with very low birth weight (VLBW, < 1500 g).

**Methods:**

Longitudinal cohort study of VLBW preterm infants from birth to early childhood. Systolic and diastolic blood pressure (SBP and DBP), and their age-, height- and sex-adjusted percentiles (SBP%, DBP%), were assessed at multiple time points. Linear quantile mixed models estimated BP trajectories across the 25th, 50th, and 75th quantiles, stratified by maternal HDP exposure.

**Results:**

Among 277 infants, 121 (43.6 %) were exposed to HDP. Median gestational age was 30 weeks (IQR: 28 - 32), and median birth weight was 1180 g (IQR: 985, 1340), 128 (46.2 %) were male. Maternal HDP was not significantly associated with SBP or DBP at any quantile. BP increased modestly with age across all quantiles. SBP increased by 0.06, 1.55, and 2.58 mmHg; DBP by 1.77, 1.80, and 3.06 mmHg at the 25th, 50th, and 75th quantiles, respectively. Conversely, SBP% and DBP% declined with age, indicating a relative downward shift in BP percentiles over time. This decline was more pronounced for DBP%, especially in the lower quantiles.

**Conclusion:**

In this cohort of VLBW preterm children, maternal HDP was associated with modest differences in blood pressure, though not statistically significant. These findings suggest that the cardiovascular effects of prenatal hypertensive exposure may be delayed, subtle, or modulated by postnatal factors. Long-term follow-up is essential to clarify these trajectories and guide early prevention efforts.

## Introduction

Hypertension during childhood has emerged as an early marker of future cardiovascular and renal disease, contributing to an increased lifetime morbidity and mortality risk [1]. Children born preterm or with very low birth weight (VLBW) are especially vulnerable, due to impaired nephrogenesis, altered vascular development, and heightened susceptibility to early-onset hypertension [1,2]. Hypertensive disorders in pregnancy (HDP) — including preeclampsia and gestational hypertension—are linked to elevated blood pressure (BP) and adverse vascular phenotypes in offspring, extending from childhood into adulthood [[3], [4], [5], [6], [7]].

Multiple cohort and population-based studies support the role of intrauterine exposure to HDP in shaping long-term cardiovascular risk. Children exposed to HDP exhibit higher systolic BP, increased arterial stiffness, and subclinical endothelial dysfunction [6,8,9]. Evidence from genetic and developmental studies further corroborates the concept of fetal programming of cardiovascular outcomes [10,11]. However, most investigations have prioritized term infants or combined heterogeneous gestational age groups, potentially obscuring mechanisms specific to preterm or VLBW neonates [1,5,[12], [13], [14]].

Despite these findings, important knowledge gaps persist regarding the BP trajectories of preterm infants exposed to HDP, particularly in the VLBW population. These infants represent a biologically distinct group with combined risks — reduced nephron number, neonatal kidney injury, and prolonged hospital exposures — that may amplify susceptibility to hypertension [1]. Moreover, few studies have examined the potential interaction between maternal cardiovascular profile and offspring outcomes across the early life course [15,16].

Given these considerations, this study aims to evaluate the association between maternal HDP and systolic BP (SBP) in preterm children with VLBW. By focusing on a high-risk neonatal cohort, this research aims to clarify the contribution of maternal HDP to early-life SBP, addressing an important gap in pediatric cardiovascular prevention.

## Methods

### Study design

This prospective, single-center cohort study included VLBW preterm infants who were evaluated at a neonatal follow-up clinic between 2019 and 2024.

### Study population

Eligible participants were children up to 10 years old with a history of preterm birth and VLBW, followed at the CeClin-UCS preterm outpatient clinic, discharged from two public hospitals. The sample was divided by maternal HDP exposure. BP assessments followed the routine preterm follow-up protocol at corrected ages of 1 and 2 years, and chronological ages of 3, 5, and 10 years.

Inclusion criteria comprised children with a history of VLBW who received neonatal intensive care in Caxias do Sul city (Brazil) or the surrounding region. Exclusion criteria were congenital heart disease, genetic syndrome, twin gestation, and gestational age (GA) above 37 weeks. Written informed consent was obtained from the participants’ legal guardians and documented in the medical records. The study was approved by the local Research Ethics Committee (CAAE: 68,863,523.8.0000.5341).

### Data collection

#### Outcome

The primary outcome was BP alterations in the offspring. BP classification followed the 2017 clinical practice guidelines, categorizing participants as normotensive, having elevated BP, or hypertensive [17]. Elevated BP was defined as systolic BP (SBP) and/or diastolic BP (DBP) at or above the 90th but below the 95th percentile for age, sex, and height. Hypertension was defined as SBP and/or DBP at or above the 95th percentile.

### Blood pressure measurement

BP measurements were systematically performed during routine outpatient follow-up visits. BP was measured on the right upper limb using an appropriately sized cuff, with the child seated comfortably. Measurements were initiated only after the child had adapted to the environment and remained calm. Three consecutive readings were obtained using a Mindray uMEC10® digital multiparameter monitor (oscillometric method, measurement range: 0–300 mmHg), and the mean value was calculated. If the mean BP value was above the normal range for age, sex, and height, confirmation was performed using the auscultatory method during the same visit. To minimize agitation and crying, which could affect the measurements, musical videos or children's books were used as distractions when necessary.

#### Exposure

Exposure was defined as the presence of any HDP, including gestational hypertension, preeclampsia, or eclampsia. Maternal hypertensive status was determined based on clinical diagnoses recorded in medical records and/or documented history of antihypertensive medication use during pregnancy. Hypertensive disorders were defined according to standard obstetric criteria, including the diagnosis of new-onset hypertension after 20 weeks of gestation with or without associated proteinuria or end-organ dysfunction (gestational hypertension, preeclampsia, or eclampsia). For the purposes of this study, all types of hypertensive disorders were analysed collectively as a single exposure group, regardless of severity, timing of onset (early vs. late), or specific clinical classification. Infants born to mothers without any history of hypertension during pregnancy were classified as unexposed.

#### Neonatal characteristics

Data on pregnancy, delivery, and neonatal outcomes during hospitalization were collected from the neonatology service database and the outpatient clinic records. Neonatal variables assessed included gestational age (GA) in weeks (determined based on early ultrasound and/or last menstrual period, and confirmed by postnatal pediatric physical examination) birth weight in grams, sex, small for gestational age (SGA; birth weight below the 10th percentile), Apgar scores at 1 and 5 min, early-onset sepsis (< 72 h post-birth), late-onset sepsis (> 72 h), retinopathy of prematurity, duration of invasive and non-invasive ventilation (in days), bronchopulmonary dysplasia (BPD; defined as the requirement for supplemental oxygen at 36 weeks' postmenstrual age).

#### Maternal characteristics

Maternal characteristics included maternal age (years), number of pregnancies, number of prenatal consultations, type of delivery (vaginal or cesarean), use of specific medications during pregnancy (captopril, enalapril, losartan, or nonsteroidal anti-inflammatory drugs), glycemic syndrome, and HDP.

### Statistical analysis

#### Sample size

Sample size calculation was based on the cohort study by Huang et al.^6^ which identified a twofold increased risk of hypertensive disease in the offspring of mothers with HDP (HR 2.11; 95 % CI 1.96–2.27). It was assumed that 25 % of the reference population in this study would experience BP alterations. To detect a relative risk of 2.0 between exposed and nonexposed groups, with a significance level of 5 % and a statistical power of 80 %, a minimum of 63 participants per group was required, yielding a total sample size of at least 126 participants.

#### Analysis

Continuous variables were expressed as median and interquartile range (IQR). Variables were compared using the Student *t*-test for quantitative data with normal distribution or the Mann-Whitney U test for ordinal or non-Gaussian data. The chi-square and Fisher's exact tests were used to assess associations between categorical variables, while the Mann-Whitney test compared medians and ordinal variables.

Systolic BP (SBP, mmHg) and systolic BP percentiles (SBP %) — adjusted for age, sex, and height — were analysed as continuous outcomes. Linear quantile mixed models (LQMM) were employed to estimate the 25th, 50th (Median), and 75th quantiles of the distribution of SBP and SBP % according to age and maternal hypertensive status. The models included SBP % and maternal hypertension (binary: hypertensive vs. normotensive pregnancy) as fixed effects, and a random intercept for each participant to account for within-subject correlation. Predicted trajectories were plotted using ggplot2. Each quantile (25th, 50th, and 75th) was displayed in a separate panel using faceting. The predicted systolic BP percentiles (SBP %) were represented on the y-axis, while age was displayed on the x-axis.

A p-value < 0.05 was considered statistically significant. Statistical analyses were conducted using R software for Windows, version 4.5.0.

## Results

### Neonatal characteristics

**Figure 1:**
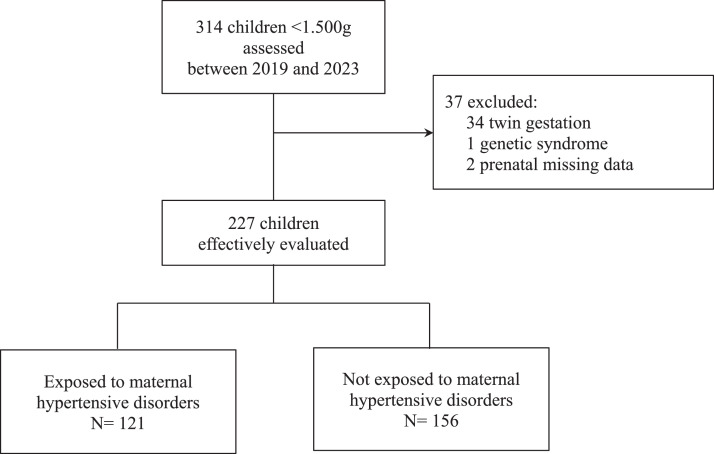
Study flow diagram.

A total of 314 VLBW infants met the eligibility criteria. Of these, 37 were excluded: 34 due to twin gestation, 1 due to a genetic syndrome, and 2 due to missing prenatal data. The final analytical sample included 277 infants, of whom 121 (43.6 %) were exposed to HDP (Figure 1).

Overall, the cohort had a median gestational age (IQR) of 30 weeks (28 - 32), and a median birth weight (IQR) of 1180 g (985, 1340). Of the 277 children, 128 (46.2 %) were male. Children exposed to maternal HDP were born at a significantly higher gestational age compared to unexposed ones (median 31 vs. 29 weeks; *p* < 0.001). The occurrence of SGA was also significantly higher in the exposed group compared to the unexposed group (47.1 % vs. 21.1 %, χ² = 20.9; *p* < 0.001). This finding is consistent with a lower birth weight z-score in the HDP group (median [IQR]:1.27 [–1.63, –0.79] vs –0.34 [–1.09, 0.27]). Additionally, this group showed a higher frequency of antenatal corticosteroid exposure, shorter duration of mechanical ventilation, and higher Apgar scores at five minutes of life. Table 1 summarizes the distribution of the study population according to exposure to maternal HDP.

**Table 1:** Neonatal and maternal characteristics.

	Exposed to HDP (121)	Non-Exposed to HDP (156)	P value
Perinatal factors			
Maternal age, Years [IQR]	29.5 [23.0,35.0]	25.0 [20.0,30.0]	<0.01
Maternal diabetes, n (%)	20 (16.6)	8 (5.1)	<0.01
Maternal body mass index [IQR]	27.24 [24.03,32.46]	25.24 [22.64,27.06]	<0.01
Maternal parity [IQR]	1 [0, 2]	1 [0,1]	0.12
Number of prenatal visits, median [IQR]	7 [5,9]	5 [4,6]	<0.01
Maternal education at childbirth			
0–9 years, n (%)	59 (48.8)	49 (31.4)	0.2
> 9 years, n (%)	32 (26.4)	41 (26.3)	0.2
Unknown	30 (24.8)	66 (42.3)	<0.01
Antenatal corticosteroid use, n (%)	73 (60.3)	61 (39.1)	0.01
Neonatal factors			
Male sex, n (%)	53 (43.8)	75 (48.0)	0.56
Gestational age, weeks [IQR]	31 [29, 32]	29 [27, 31]	<0.01
< 28 weeks, n (%)	12 (10.1)	32 (20.5)	<0.01
Birth weight, g [IQR]	1200 [1000, 1350]	1167 [962,1321]	0.47
< 1000 g, n (%)	28 (23)	45 (29)	0.35
Birth weight z score [IQR]	−1.27 [−1.63, −0.79]	−0.34 [−1.09, 0.27]	<0.01
Small for gestational age, n (%)	57 (47.1)	33 (21.1)	<0.01
SNAPPE-II SCORE [IQR]	8 [0, 20]	15 [5, 24]	0.05
Apgar score (5 min) [IQR]	9 [8,9]	8 [7,9]	<0.01
Ventilation (days), median [IQR]	0 [0, 3]	2 [0, 6]	<0.01
Length of stay (days), median [IQR]	39.5 [31.0, 55.0]	52.5 [40.0, 76.5]	<0.01
Blood pressure			
Median systolic blood pressure [IQR]	100 [93, 108]	99 [92, 105]	0.32
Median diastolic blood pressure [IQR]	63 [58, 70]	60 [55, 67]	0.03
High blood pressure (≥ P90 and < P95)	37 (30.5)	42 (26.9)	0.59
Hypertension (≥ P95)	54 (44.6)	59 (37.8)	0.31

Values are presented as numbers with percentages in parentheses or as medians with interquartile ranges (IQR) in square brackets, as appropriate. SNAPPE-II: Score for Neonatal Acute Physiology Perinatal Extension II.

### Maternal characteristics

Maternal age was significantly higher among mothers with HDP (median 29.5 years; IQR 23.0–35.0) compared to normotensive mothers (median 25.0 years; IQR 20.0–30.0; *p* < 0.01). Maternal gestational diabetes was more frequent among mothers with HDP (16.7 %) than among normotensive mothers (5.3 %). (*p* < 0.01). Maternal body mass index (BMI) at the beginning of pregnancy was significantly higher in mothers with HDP (median 27.24 kg/m²; IQR 24.03–32.46) compared to normotensive mothers (median 25.24 kg/m²; IQR 22.64–27.06; *p* < 0.01) (Table 1).

### Longitudinal quantile regression for systolic and diastolic blood pressure (mmHg)

Table 2 presents the quantile regression estimates for SBP and DBP at the 25th, 50th, and 75th quantiles. The intercept increased progressively across quantiles, reflecting higher baseline SBP or DBP at higher quantiles. Maternal hypertension was not significantly associated with SBP at any quantile. The mean differences in SBP between exposed and unexposed groups were minimal across quantiles: 0.06 mmHg higher in the exposed group at the 25th quantile, 1.55 mmHg at the 50th quantile, and 2.58 mmHg at the 75th quantile, none of which reached statistical significance. Age showed a positive association with SBP, reaching statistical significance only at the 75th percentile (β = 1.04; 95 % CI 0.17–1.90; *p* = 0.02). For DBP, the mean differences between exposed and unexposed groups across quantiles were as follows: 1.77 mmHg higher at the 25th quantile, 1.80 mmHg at the 50th quantile, and 3.06 mmHg at the 75th quantile, with the latter reaching statistical significance (β = 3.06; 95 % CI 0.06–6.05; *p* = 0.04).

**Table 2:** Fixed effects estimates from linear quantile mixed models (LQMM) predicting systolic and diastolic blood pressure (mmHg) at the 25th, 50th, and 75th quantiles.

		Systolic Blood Pressure (mmHg)				Diastolic Blood Pressure (mmHg)			
Quantile	Variable	Estimate	Std. Error	95 % CI	p-value	Estimate	Std. Error	95 % CI	p-value
0.25	(Intercept)	90.79	1.46	(87.84, 93.73)	<0.01	54.21	1.17	(51.86, 56.74)	<0.01
	Age	0.69	0.38	(–0.07, 1.45)	0.07	0.38	0.26	(−0.15, 0.91)	0.15
	Maternal Hypertension	0.06	1.44	(–2.84, 2.96)	0.96	1.77	1.36	(−0.96, 4.50)	0.19
0.50	(Intercept)	95.52	1.50	(92.51, 98.53)	<0.01	59.19	1.32	(56.52, 61.85)	<0.01
	Age	0.90	0.45	(–0.00, 1.80)	0.05	0.39	0.36	(−0.33, 1.11)	0.28
	Maternal Hypertension	1.55	1.55	(–1.57, 4.67)	0.32	1.80	1.18	(−0.58, 4.17)	0.13
0.75	(Intercept)	100.81	1.65	(97.49, 104.13)	<0.01	65.29	1.51	(62.25, 68.32)	<0.01
	Age	1.04	0.43	(0.17, 1.90)	0.02	0.41	0.29	(−0.18, 1.00)	0.17
	Maternal Hypertension	2.58	1.49	(–0.42, 5.58)	0.09	3.06	1.49	(0.06, 6.05)	0.04

### Longitudinal quantile regression for systolic and diastolic blood pressure percentiles

The longitudinal quantile regression analysis estimated the association between SBP % and DBP % across age, stratified by maternal hypertensive status, at the 25th, 50th, and 75th quantiles (Table 3). The model intercepts represent the estimated SBP % and DBP % at birth (theoretical age zero) among children born to normotensive mothers.

**Table 3:** Fixed effects estimates from linear quantile mixed models (LQMM) predicting systolic and diastolic blood pressure percentiles at the 25th, 50th, and 75th quantiles.

		Systolic Blood Pressure Percentile (SBP %)				Diastolic Blood Pressure Percentile (DBP %)			
Quantile	Variable	Estimate	Std. Error	95 % CI	p-value	Estimate	Std. Error	95 % CI	p-value
0.25	(Intercept)	73.76	3.14	(67.45, 80.07)	<0.01	95.87	1.88	(92.09, 99.66)	<0.01
	Age	−3.35	1.00	(−5.35, −1.34)	<0.01	−5.92	0.62	(−7.18, −4.66)	<0.01
	Maternal Hypertension	0.04	3.61	(−7.22, 7.30)	0.99	3.34	2.12	(−0.92, 7.62)	0.12
0.50	(Intercept)	87.00	2.72	(81.52, 92.47)	<0.01	99.82	2.02	(95.75, 103.91)	<0.01
	Age	−1.85	0.90	(−3.66, −0.05)	0.04	−4.04	0.99	(−6.04, −2.05)	<0.01
	Maternal Hypertension	1.96	2.92	(−3.90, 7.82)	0.505	2.56	1.69	(−0.84, 5.96)	0.13
0.75	(Intercept)	96.44	0.91	(94.61, 98.27)	<0.01	101	1.04	(99.50, 103.71)	<0.01
	Age	−0.45	0.25	(−0.95, 0.04)	0.073	−2.25	0.52	(−0.94, 2.93)	<0.01
	Maternal Hypertension	0.80	1.20	(−1.61, 3.22)	0.508	0.99	0.96	(−0.94, 2.93)	0.30

Note: 95 % confidence intervals are reported. SBP % and DBP % represents individual systolic and diastolic blood pressure percentiles for age, sex, and height.

Across all quantiles, age was negatively associated with SBP % and DBP %, indicating a decline in percentiles with increasing age. This association was statistically significant in all quantiles. The magnitude of the age-related decline was substantially greater for DBP % compared to SBP %, particularly at lower quantiles. This suggests a steeper downward trajectory in diastolic percentiles over time. The quantile-specific coefficients for age were markedly more negative in the DBP % models, indicating a stronger inverse association between age and DBP %, especially among children born to normotensive mothers. Maternal hypertensive status was not significantly associated with systolic BP percentiles at any quantile (Table 3).

### Predicted trajectories of systolic and diastolic blood pressure percentiles

**Figure 2:**
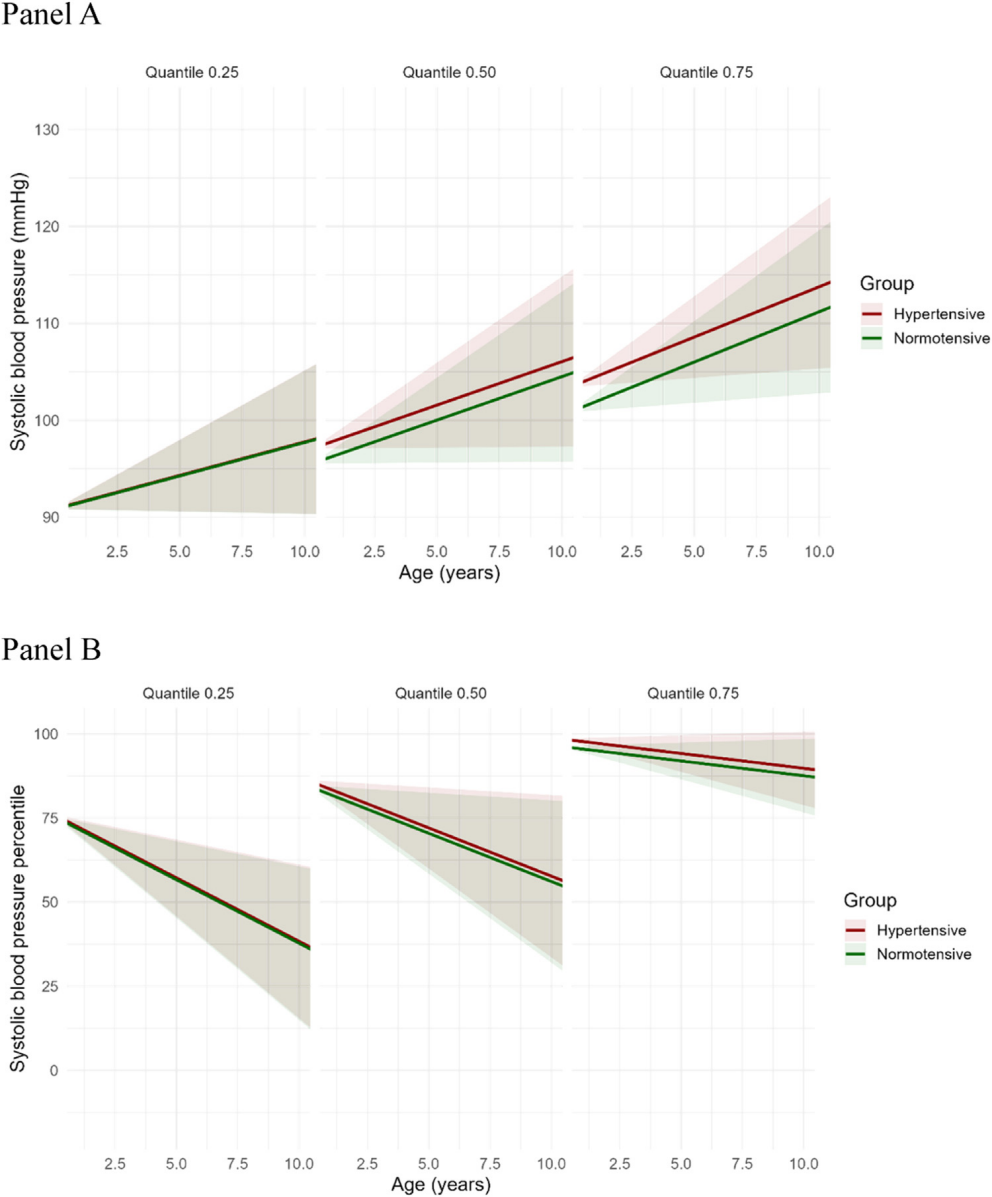
Predicted trajectories of systolic blood pressure (mmHg, SBP, Panel A) and SBP percentiles (SBP %, Panel B) according to maternal hypertensive status. Each panel displays a quantile (25th, 50th [Median], or 75th) of the distribution of SBP. Panel A shows the absolute values of systolic blood pressure (SBP, in mmHg), which increases with age across all quantiles. The mean differences in SBP between exposed and unexposed to hypertensive disorders were minimal across quantiles: 0.06 mmHg higher in the exposed group at the 25th quantile, 1.55 mmHg at the 50th quantile, and 2.58 mmHg at the 75th quantile. Panel B depicts SBP expressed as percentiles (SBP %), which decrease with increasing age. The rate of decline is steeper at the 25th quantile, intermediate at the 50th quantile, and more gradual at the 75th quantile, indicating quantile-dependent variation in the trajectory of SBP % over time. Red and green lines represent hypertensive and normotensive groups, respectively. Shaded areas represent 95 % confidence intervals. Panel APanel B.

**Figure 3:**
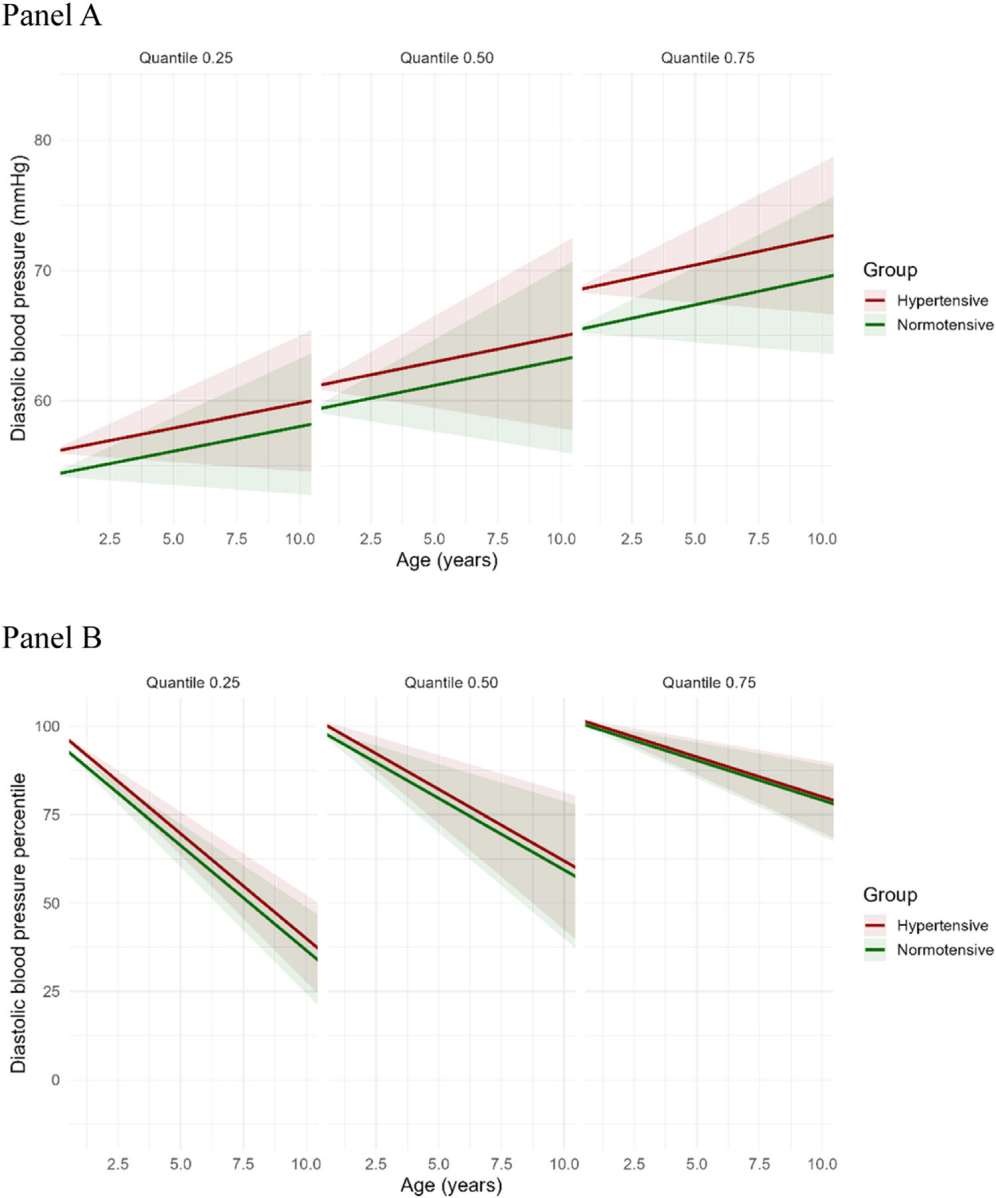
Predicted trajectories of diastolic blood pressure (mmHg, DBP, Panel A) and DBP percentiles (DBP %, Panel B) according to maternal hypertensive status. Each panel displays a quantile (25th, 50th [Median], or 75th) of the distribution of DBP. Panel A shows the absolute values of diastolic blood pressure (DBP, in mmHg), which increases with age across all quantiles. The mean differences in DBP between exposed and unexposed to hypertensive disorders were small across quantiles: 1.77 mmHg higher in the exposed group at the 25th quantile, 1.80 mmHg at the 50th quantile, and 3.06 mmHg at the 75th quantile. Panel B depicts DBP expressed as percentiles (DBP %), which decrease with increasing age. The rate of decline is steeper at the 25th quantile, intermediate at the 50th quantile, and more gradual at the 75th quantile, indicating quantile-dependent variation in the trajectory of DBP % over time. Red and green lines represent hypertensive and normotensive groups, respectively. Shaded areas represent 95 % confidence intervals. Panel APanel B.

The predicted trajectories of SBP, DBP, SBP %, and DBP % demonstrated distinct patterns across age and quantiles of the distribution, stratified by maternal hypertensive status (Panel A in Figures 2 and 3). When expressed as absolute values (mmHg), both SBP and DBP increased with age across all quantiles (25th, 50th, and 75th). The average differences in SBP and DBP between children exposed and unexposed to maternal HDP were modest, suggesting a slight upward shift in blood pressure among exposed children, more pronounced at higher quantiles. An upward displacement of the absolute SBP and DBP values (mmHg) is visually evident in the graphical representation, particularly at the 75th quantile (Figures 2 and 3).

In contrast, when SBP and DBP were expressed as percentiles (SBP %, DBP %), a consistent decline with increasing age was observed. The rate of decline was steepest at the 25th quantile, intermediate at the 50th quantile, and more gradual at the 75th quantile, indicating a quantile-dependent variation in the trajectory of SBP % and DBP % over time (Panel B in Figures 2 and 3). These results imply that although SBP and DBP values tend to rise with age, their relative position within the population distribution diminishes, especially among those in the lower percentiles. This downward trend was more pronounced for DBP %, suggesting a steeper decline in diastolic pressure percentiles compared to systolic percentiles across childhood.

## Discussion

This study examined the association between maternal HDP and longitudinal BP outcomes in 277 VLBW infants. The primary findings include modest differences in SBP and DBP trajectories across childhood, a progressive decline in SBP % and DBP % over time, and a higher frequency of SGA births among offspring of hypertensive mothers.

These findings align with a growing body of evidence suggesting that intrauterine exposure to maternal HDP is associated with modest but persistent elevations in BP across the lifespan [5,14,[18], [19], [20]]. Recent findings underscore the cumulative cardiometabolic influence of maternal conditions—including HDP—on offspring cardiovascular health [5]. Consistent with this, Alsnes et al. reported sustained increases in SBP and DBP during childhood and adolescence in offspring of hypertensive pregnancies [18]. Similarly, Davis et al. observed a mean increase of 2.39 mmHg in SBP and 1.35 mmHg in DBP among exposed individuals in a meta-analysis, supporting the hypothesis that even minor BP elevations in early life may contribute to lifelong cardiovascular risk [18]. The authors’ own finding of a ∼2 mmHg difference at the upper SBP quantile and a ∼3 mmHg difference at the upper DBP quantile is in line with this evidence and may reflect early manifestations of developmental programming. Although modest in magnitude, these elevations are clinically relevant, as BP in childhood tracks into adulthood and predicts future cardiovascular outcomes [21].

These results also underscore the importance of distribution-sensitive approaches — such as quantile regression — to detect risk heterogeneity across the SBP and DBP distribution, which may be masked in mean-based analyses. Importantly, all children in the studied cohort were born VLBW, a population inherently predisposed to elevated BP and cardiovascular morbidity. This uniform high-risk background may have attenuated between-group differences, potentially masking additional contributions of maternal HDP. The co-occurrence of HDP and prematurity warrants particular attention, as both are independently associated with increased cardiovascular risk in offspring. However, separating their individual and combined effects remains a challenge. Nahum Sacks et al. highlighted the importance of clarifying whether HDP amplifies the cardiovascular risk conferred by prematurity, or vice versa [22]. Similar concerns were noted in prior research, where low birth weight, adiposity, and other perinatal factors confounded associations between maternal HDP and offspring BP [3,18,23].

Moreover, most prior studies evaluated outcomes in adolescents or young adults, when cardiovascular phenotypes are more fully expressed [3,5,19]. In contrast, the present study focuses on early childhood — a critical but underexplored window when BP trajectories may still be modifiable. Supporting this, Gootjes et al. reported no significant association between maternal HDP and SBP or DBP in children under six years [16]. Data from an Indian cohort revealed elevated SBP, but not DBP, at ages 3–7 years among those exposed to HDP.⁸ A Mendelian randomization study by Wang et al. found no evidence of a causal effect of maternal BP on offspring cardiometabolic health, including BP, using data from over 29,000 mother-offspring pairs. Together, these results suggest that intrauterine effects on offspring BP may be subtle or latent in early childhood, emphasizing the importance of long-term follow-up [24].

In the studied cohort, absolute SBP and DBP values increased with age — consistent with growth and maturation. However, SBP % and DBP % declined over time, especially among individuals in the lower quantiles — highlighting a dynamic, quantile-dependent BP trajectory. These age- and distribution-sensitive trends reinforce the need to interpret childhood BP against population-based norms. Despite these nuanced patterns, the longitudinal quantile regression did not reveal a significant association between maternal HDP and either SBP or DBP across age or quantiles. This contrasts with Hovi et al., who found significantly higher BP in young adults born VLBW compared to those born at term. Among individuals exposed to maternal HDP, the elevation in BP was even greater, suggesting that the effects of prenatal HDP exposure on BP may become more apparent later in life [2].

Several factors may explain the absence of significant associations in the present study. In addition to the homogeneous VLBW profile, which itself elevates cardiovascular risk,^2^ advances in perinatal care may have mitigated intrauterine HDP effects. Postnatal environments also play a crucial role: Meng et al. demonstrated that BP in early life is fluid, with trajectories influenced by growth, behavior, and socioeconomic conditions [25]. These findings emphasize the need for a life-course approach, as postnatal factors may modify or delay phenotypic expression of prenatal exposures [25].

The high frequency of SGA among exposed infants aligns with established associations between HDP and impaired fetal growth [2,[26], [27], [28]]. However, in the present cohort, no significant differences in BP values were observed between SGA and AGA children. This finding is consistent with the results reported by Hovi et al., who found that both SGA and AGA individuals born at VLBW had similarly elevated SBP and DBP compared to term-born controls, suggesting that prematurity and VLBW themselves may be the primary contributors to altered BP, regardless of fetal growth status [2].

A major strength of this study is the longitudinal analysis of a well-characterized VLBW cohort, using quantile-specific a method that captures variations in BP distribution often missed by conventional means. However, its single-center design may limit external validity. Residual confounding cannot be fully excluded, including lifestyle, genetic, and postnatal factors, or variability in adherence. These aspects should be considered when interpreting the results.

In this high-risk VLBW cohort, maternal HDP was not significantly associated with early childhood blood pressure. These findings suggest that the cardiovascular effects of prenatal hypertensive exposure may be delayed, subtle, or overshadowed by postnatal factors. Longitudinal monitoring into adolescence and beyond is essential to fully capture the trajectory of risk and guide early preventive strategies.

## Funding

This research did not receive any specific grant from funding agencies in the public, commercial, or not-for-profit sectors.

## Data availability

Data that support the findings of this study are available from the corresponding author.

## Conflicts of interest

The authors declare no conflicts of interest.
